# The Development and Evaluation of a Combined Infection–Rheumatology Assessment Service in Response to the Chikungunya Fever Epidemic

**DOI:** 10.4269/ajtmh.22-0698

**Published:** 2023-03-20

**Authors:** Maria Krutikov, Joseph Donovan, Jonathan Lambourne, Coziana Ciurtin, Mike Brown, Robin Bailey, Jessica J. Manson

**Affiliations:** 1 Department of Rheumatology, University College London Hospitals NHS Trust, London, United Kingdom;; 2 Hospital for Tropical Diseases, University College London Hospitals NHS Trust, London, United Kingdom;; 3 Clinical Research Department, London School of Hygiene and Tropical Medicine, London, United Kingdom;; 4 Centre for Adolescent Rheumatology, Division of Medicine, University College London, London, United Kingdom

## Abstract

The chikungunya virus is an arthritogenic alphavirus. Acute infection may be followed by persistent arthralgia, often causing significant functional impairment. The 2014–2015 chikungunya fever (CHIKF) epidemic resulted in a marked increase in cases presenting to rheumatology and tropical diseases services. A combined multidisciplinary rheumatology–tropical diseases service for assessment, management, and follow-up of patients with proven CHIKF and persistent (≥ 4 weeks) arthralgia was proposed and rapidly developed at The Hospital for Tropical Diseases in London. Rapid set up of a multidisciplinary clinic in response to the epidemic was achieved. Of a total of 54 patients, 21 (38.9%) patients with CHIKF developed persistent arthralgia and were reviewed by the multidisciplinary service. A combined assessment approach enabled comprehensive multidisciplinary assessment of CHIKF, assessment of joint pathology through ultrasound, and appropriate follow-up. A combined rheumatology–tropical diseases service was successfully used to identify and assess CHIKF-associated morbidity. Future outbreaks may be approached by establishing tailored multidisciplinary clinics.

Chikungunya fever (CHIKF) is an acute viral febrile illness characterized by severe polyarthralgia/polyarthritis, myalgia, headache, and rash.^1^ Infection occurs when the chikungunya virus (CHIKV)—an RNA alphavirus of the Togaviridae family—is transmitted to humans via the bite of an infected *Aedes* mosquito. The term “chikungunya” is taken from the Makonde language, where it means “that which bends up,”^2^^,^^3^ referring to the posture taken up by individuals incapacitated by the extreme joint pains of severe disease.^4^ Persistent, often disabling, arthralgia is common. Many patients experienced prolonged rheumatological disease lasting for months or sometimes years.^5^^,^^6^ Chikungunya virus was first identified during an epidemic in Tanzania in 1952. However, in late 2013 a dramatic expansion of cases occurred, with ∼1.7 million infections reported from 45 countries in the Americas between December 2013 and September 2015.^7^ Chikungunya fever outbreaks were reported in the Pacific Islands in 2014, with this same year witnessing Europe’s highest CHIKF burden with almost 1,500 cases.^8^ Locally acquired CHIKV transmission has now been reported in 114 countries in territories inhabited by three-quarters of the world’s population, with disabling effects of CHIKF disproportionately affecting the poorest communities.^9^^,^^10^ Further geographical expansion of *Aedes*-transmitted viruses, including CHIKV, is expected in the context of climate change,^11^ and future service planning will need to adapt to new epidemics accordingly. Following the 2013 CHIKF outbreak in the Americas, The Hospital for Tropical Diseases (HTD) in London saw a sudden increase in returning travelers with significant joint pain consistent with recent CHIKF. A combined rheumatology–tropical diseases service for assessment, management, and follow-up of these patients was proposed to meet clinical need and facilitate a multidisciplinary, single-visit approach to clinical assessment of patients with persistent arthralgia due to CHIKF. Here, we describe the development of this combined rheumatology–tropical diseases assessment service, assess the extent of CHIKF pathology and morbidity in patients with CHIKF at HTD, and describe long-term follow-up.

The combined rheumatology–tropical diseases service was developed and supported by rheumatology and tropical diseases consultants at University College London Hospitals (UCLH) NHS Foundation Trust in response to the CHIKF epidemic. A clinical management guideline for CHIKF was written to guide history taking, key examination points, a testing panel, and management options. Initially, patients with CHIKF, defined as either a positive polymerase chain reaction result or a serological profile suggestive of acute infection (elevated IgM and/or significantly elevated IgG) in the presence of a consistent travel history, presenting to the HTD were booked for review. The service was also advertised externally to local general practitioners via social media. After initial assessment, patients with symptoms for ≥ 4 weeks were offered an appointment with a combined rheumatology–tropical diseases assessment service. Combined clinic assessment was undertaken by both rheumatology and tropical diseases specialists, enabling appropriate assessments, including musculoskeletal ultrasound to be performed at a single visit. Baseline data were collected, including demographics (date of birth, age, sex), travel details, presenting symptoms, time from referral to review, relevant blood tests, musculoskeletal ultrasound findings, and use of therapies. Testing for imported viruses (CHIKV, dengue) was carried out in the United Kingdom’s Rare and Imported Pathogens Laboratory. Ultrasound examination of the hands and/or feet was performed as appropriate for the patient’s symptoms using a Logiq S8 US machine (GE Medical Systems Ultrasound and Primary Care Diagnostics, Wauwatosa, WI). Patients were followed up either by telephone or clinic visit. Additional follow-up was performed in 2017—to assess whether arthralgia had resolved, was improving, was persistent , or had worsened—with a final review of follow-up electronic records in 2022. Data were summarized using median values (with first and third quartiles) for continuous data and the number (with frequency [%]) for categorical data and analyzed in Microsoft Excel. This service evaluation project was approved by and registered with UCLH NHS Foundation Trust. As an evaluation of a local clinical service, this is not considered research, and patient consent for this project was not required. Patient-reported data describing instances where patient numbers were fewer than five were not included, in line with UCLH Information Governance Department advice.

Between March 2014 and January 2015, 54 patients with laboratory-diagnosed CHIKF were evaluated at HTD. A subgroup of CHIKF patients with persistent (≥ 4 weeks) arthralgia (21 of 54 [39%]) was assessed in the combined rheumatology–tropical diseases clinic. Baseline characteristics are described in Table 1. Most individuals acquired CHIKV from the Caribbean region. Overall, 17 of 54 (31.5%) patients acquired CHIKV from travel to Jamaica (versus 12 of 21 [57.1%] in the persistent arthralgia subgroup).

**Table 1:** Chikungunya at The Hospital for Tropical Diseases

Characteristic	All CHIKF patients*		Persistent arthralgia†	
	*N*	Summary statistic‡	*N*	Summary statistic
Age, years	54	54 (39, 62)	21	58 (51, 65)
Sex, *n* (%)				
Male	54	19 (35.2)	21	6 (28.6)
Female		35 (64.8)		15 (71.4)
Selected symptoms, *n* (%)				
Fever	53	39 (73.6)	21	14 (66.7)
Arthralgia		53 (100)		21 (100)
Rash		28 (52.8)		13 (61.9)
Positive CHIKV testing, *n* (%)				
IgM	49	12 (24.8)	21	4 (19.0)
IgG	49	47 (95.9)	21	21 (100)
PCR	36	4 (11.1)	13	2 (9.5)
CRP	52	2.3 (0.8, 5.9)	20	2.4 (1.0, 5.4)
ESR	44	10.5 (8.0, 16.0)	21	10.5 (8.0, 16.0)

CHIKV = chikungunya virus; CRP = C-reactive protein; ESR = erythrocyte sedimentation rate; PCR = polymerase chain reaction.

* “All patients” refers to patients with laboratory-diagnosed CHIKF presenting to The Hospital for Tropical Diseases between August 2014 and January 2015.

† “Persisting arthralgia” refers to those patients with persistent arthralgia, defined as symptoms for at least 4 weeks, who were subsequently seen by the combined rheumatology–tropical diseases assessment service. Patients with persisting arthralgia are a subgroup of “all patients”.

‡ Summary statistic is the median (first and third quartile) value for continuous data and the number and frequency (%) of patients with the characteristic for categorical data.

In the subgroup with persistent arthralgia, the median time between symptom onset and specialist clinic review was 12.1 weeks. Further characterization of the persistent arthralgia subgroup, including ultrasound findings, is shown in Table 2. In patients undergoing joint ultrasound, synovial hypertrophy and joint effusion were found in 17 of 19 (89.5%) and 18 of 19 (94.7%) patients, respectively. Ultrasound findings correlated well with symptoms. Tests for alternative infectious diagnoses were negative in all patients with persistent arthralgia. A positive rheumatoid factor was found in 5 of 21 (23.8%) patients, with only one at high titer (range: 14–242 IU/mL). No patients had positive anti-nuclear antigen or anti-cyclic citrullinated peptide (anti-CCP) antibodies. Overall, intramuscular or oral corticosteroids were used as therapy in 6 of 54 (11.1%) patients, whereas nonsteroidal anti-inflammatory drugs were used in 34 of 54 (63.0%) patients.

**Table 2:** Further characterization and ultrasound findings of patients with persistent arthralgia at the combined rheumatology–tropical diseases assessment service

Characteristic	Patients	
	*N*	*n* (%)
Number of tender joints per patient out of 28 (DAS28* examination),† *n*	21	2 (0, 5)
Swollen joints per patient out of 28,† *n*	21	0 (0, 2)
Global VAS†	21	40 (20, 70)
DAS28-CRP	20	3.11 (2.05, 4.04)
Joints affected symptomatically,† *n* (%)		
Hand	21	12 (57.1)
Wrist		10 (47.6)
Knee		15 (71.4)
Ankle		12 (57.1)
Feet		13 (61.9)
Joint involved on ultrasound, *n* (%)		
Hand	19	9 (47.4)
Wrist		8 (42.1)
Ankle		3 (15.8)
Feet		7 (36.8)
Ultrasound findings,‡ *n* (%)		
Synovial hypertrophy	19	17 (89.5)
Synovial hypertrophy ≥ grade 2	19	9 (47.4)
Joint effusion	19	18 (94.7)
Osteophytes	19	9 (47.4)
Positive Power Doppler	19	3 (15.8)
Positive Power Doppler > 1+	19	0 (0)
ANA, *n* (%)		
Negative	21	19 (28.6)
Weak positive		2 (71.4)
Anti-CCP, units/mL (%)		
Negative	21	21 (100)
RhF, IU/mL (%)		
Negative	21	15 (71.4)
Positive§		6 (28.6)

ANA = anti-nuclear antigen; CCP = cyclic citrullinated peptide; DAS = disease activity score; *N* = number of patients included in that statistic; RhF = rheumatoid factor; VAS = visual analogue score (patient's assessment of the global impact of disease on life). “Persistent arthralgia” refers to patients with arthralgia for at least 4 weeks who were subsequently seen by the combined rheumatology–tropical diseases assessment service. Ultrasound was performed in 19 of 21 patients seen in the combined rheumatology–tropical diseases assessment service.

* The disease activity score (DAS)28-CRP was used to assess the extent of joint involvement in each patient (http://www.das-score.nl).^18^ DAS28-CRP was chosen given the absence of a validated scale specifically for the assessment of chikungunya virus (CHIKF)-associated arthralgia disease severity and the resemblance of the pattern of joint involvement in CHIKF to that of rheumatoid arthritis.^12^^,^^19^ For DAS28-CRP, a score > 2.6 indicates low disease severity, a score > 3.2 indicates moderate disease severity, and a score > 5.1 indicates high disease severity.

† Evaluated by clinical assessment.

‡ Evaluated by ultrasound assessment. Synovial hypertrophy ≥ grade 2 indicates an increased severity of synovial hypertrophy (grade 2 = moderate, grade 3 = severe). Summary statistic is the median (first and third quartile) value for continuous data, and the number and frequency (%) of patients with the characteristic for categorical data.

§ Includes any positive (values, listed in order of magnitude: 241.8, 19.8, 18.4, 18.3, 17.5, 16.6).

Telephone or clinic follow-up was performed in 45 of 54 (83.3%) patients, including all 21 patients with persistent arthralgia seen in the combined clinic. First follow-up was performed at a median of 6.3 weeks (range: 1–22 weeks). In the persistent arthralgia subgroup, symptoms of 14/21 (66.7%) patients had resolved or were improving. No patients deteriorated. Further telephone follow-up was undertaken with the persistent arthralgia subgroup at a median of 31 months (range: 29–33 months). At this time 7 of 21 (33.3%) patients were asymptomatic, 9 of 21 (42.9%) patients had mild or severe ongoing symptoms, and 5 of 21 (23.8%) patients were lost to follow-up. For individuals requiring subsequent UCLH rheumatology follow-up from 2015 to 2022, there was a median of 3.25 appointments per patient per year (interquartile range: 1.8–5.0).

A combined rheumatology–tropical diseases service for assessment, management, and follow-up of patients with CHIKF and persistent arthralgia was proposed and rapidly established at the HTD in London in response to the 2014–2015 CHIKF epidemic. Patients were assessed by multidisciplinary clinical assessment, including careful characterization of joint disease by ultrasound, at a single visit. Despite the relatively large numbers of individuals affected by persistent arthralgia in CHIKF, descriptions of ultrasound features are uncommon.^12^^–^^14^ In patients with persistent arthralgia, synovial hypertrophy and joint effusion were common. Demonstration of joint effusion is consistent with a description of ankle ultrasound features in a Brazilian CHIKF cohort.^15^ In our study both synovial hypertrophy and joint effusion were extremely common, although synovial hypertrophy was graded 2 (on a scale of 0–3, with grade 2 being moderate synovial hypertrophy) or higher in fewer than 50% of patients. Our findings suggest that pathophysiology in CHIKF is very different from that of rheumatoid arthritis. Recent papers have suggested variation in clinical manifestations according to CHIKV genotype, with more erosive disease seen in the East Central/South American genotype as opposed to the Asian genotype, the strain associated with the Caribbean epidemic.^16^^,^^17^ This may account for the lack of erosions seen in our cohort who had largely traveled to the Caribbean. In our study, most patients had persistent symptoms at follow-up, but only a minority had recalcitrant symptoms requiring systemic steroids or prolonged follow-up with rheumatology services. Few individuals remained in ongoing rheumatology follow-up 7 years after their initial diagnosis.

There are limitations to this evaluation. We only identified individuals who were referred or who self-presented to the HTD. This is certainly an underestimate of the total number of cases in the area, and persistent symptoms may be over-represented in those referred because patients with self-limiting disease would be less likely to seek help. The DAS28 was of limited utility in this cohort, with most patients scoring in the “low activity” range despite significant functional impairment and discomfort. This may have been due to the high proportion of patients with involvement of the feet and ankles; these sites were not included in the joint counts.

A strength of this project is that it shows how a dedicated combined specialty service can be rapidly established, which has value for consideration of future services in response to emerging and epidemic infectious diseases. Additionally, this project describes a comparatively large cohort of individuals with CHIKF presenting during the 2014–2015 epidemic in Europe, with persistent disease characterized by ultrasound.

In summary, we developed a combined rheumatology–tropical diseases service in response to the 2014–2015 CHIKF epidemic. Although the frequency of CHIKF cases presenting to the HTD has now decreased, the links and knowledge established through this multidisciplinary pathway have been maintained and surge clinics could be re-established in the context of new epidemics. Ultrasound was a useful adjunct to clinical assessment. Multidisciplinary clinic assessment allowed disease-tailored assessment and management in response to an epidemic.
